# Correlation between kidney transplantation and colorectal cancer in hemodialysis patients: A nationwide, retrospective, population-based cohort study

**DOI:** 10.1186/s12885-019-6283-z

**Published:** 2019-11-16

**Authors:** Han-En Wang, Yu-Chan Liao, Je-Ming Hu, Wen-Chih Wu, Wan-Yun Chou, Yong-Chen Chen, Yu-Ching Chou, Chi-Feng Hung, Yu-Feng Tian, San-Lin You, Chien-An Sun

**Affiliations:** 1 Division of Nephrology, Department of Medicine, Tri-Service General Hospital, National Defense Medical Center, Taipei City, Taiwan; 2 School of Public Health, National Defense Medical Center, Taipei City, Taiwan; 3 Graduate Institute of Medical Sciences, National Defense Medical Center, Taipei City, Taiwan; 4 Division of Colorectal Surgery, Department of Surgery, Tri-Service General Hospital, National Defense Medical Center, Taipei City, Taiwan; 5 Department of Surgery, Suao and Yuanshan branches of Taipei Veterans General Hospital, Yilan County, Taiwan; 6 Graduate Institute of Life Sciences, National Defense Medical Center, Taipei City, Taiwan; 7 Department of Medicine, College of Medicine, Fu-Jen Catholic University, New Taipei City, Taiwan; 8 Big Data Research Center, College of Medicine, Fu-Jen Catholic University, New Taipei City, Taiwan; 9 Division of Colorectal Surgery, Department of Surgery, Chi-Mei Medical Center, Tainan City, Taiwan; 10 Department of Health & Nutrition, Chia Nan University of Pharmacy and Science, Tainan City, Taiwan; 11 Department of Public Health, College of Medicine, Fu-Jen Catholic University, New Taipei City, Taiwan

**Keywords:** Colorectal cancer, Hemodialysis, Kidney transplantation, Retrospective cohort study

## Abstract

**Background:**

Kidney transplantation (KT) correlates with an increased risk of developing several malignancies; however, the risk of colorectal cancer (CRC) after KT remains debatable and has been marginally explored. Hence, in this nationwide, retrospective, population-based cohort study, we aimed to examine the correlation between KT and CRC in a large-scale population-based Chinese cohort.

**Methods:**

We identified a total of 3739 regular hemodialysis patients undergoing KT (exposed cohort) and 42,324 hemodialysis patients not undergoing KT (non-exposed cohort) between 2000 and 2008 from Taiwan’s National Health Insurance Research Database (NHIRD). Both cohorts were followed up from January 1, 2000, to the date of CRC diagnosis, death, or the end of 2013. Using Kaplan–Meier method, we measured the cumulative incidence of CRC in each cohort. Furthermore, Cox proportional hazards models were used to compute hazards ratios (HRs) and 95% confidence intervals (CIs) to estimate the correlation between KT and CRC in hemodialysis patients.

**Results:**

The Kaplan–Meier analysis revealed that the cumulative incidence of CRC was significantly higher in the exposed cohort than in the non-exposed cohort (log-rank test, *P* < 0.001). After adjusting for potential confounders, the exposed cohort exhibited a significantly increased risk of CRC compared with the non-exposed cohort (adjusted HR, 1.34; 95% CI, 1.11–1.62).

**Conclusions:**

Hemodialysis patients undergoing KT have a significantly higher risk of CRC than those not undergoing KT. Cancer should continue to be a primary focus of prevention during KT.

## Background

Kidney transplantation (KT) correlates with an increased risk of developing several malignancies. The overall incidence of cancer in kidney transplant recipients is estimated to be 1.9–18% [1–5]. Established evidence from registry data and observational studies have reported a 2.5- to 3-fold increase in the overall risk of cancer among kidney transplant recipients [1, 2]. Furthermore, the risk exponentially increases with virus-associated neoplasms such as human papillomavirus-related urogenital cancers, human herpesvirus 8-associated Kaposi’s sarcoma, and Epstein–Barr virus-related post-transplant lymphoproliferative disease, with an excess risk of, at least, 5–30 times higher than that in age- and sex-matched general population [4]. Perhaps, immunosuppression following transplantation is a critical factor in the augmented incidence of malignancy [6, 7]. The risk of carcinoma increases with cumulative and prolonged use of immunosuppressant agents [6, 7]. Advancements in surgical techniques and immunosuppressive therapies have led to enhanced survival rates of patients and grafts; thus, post-transplantation cancer development could be a critical cause of morbidity and mortality in these patients in the future.

Globally, colorectal cancer (CRC) is a leading cause of morbidity and mortality, accounting for > 9% of all cancer incidences. Reportedly, CRC is the third leading cancer worldwide and the fourth leading cause of cancer-related mortality [8]. Most studies have reported an elevated risk of CRC among solid organ transplant recipients relative to the general population, with standardized incidence ratios (SIRs) ranging from no correlation to a 4.5-fold increase [1–5]; while a meta-analysis reported an overall SIR estimate of 1.69 [9]. Nevertheless, the risk of CRC after KT remains debatable and has been marginally explored. In addition, CRC has never been reported to be caused by a viral infection, and CRC risk is not high among human immunodeficiency virus-infected individuals who are also immunosuppressed, [9] increasing the likelihood of a different underlying biological mechanism. In Taiwan, end-stage renal disease (ESRD) causes substantial medical and economic burden [10]. Reportedly, KT is usually performed for ESRD and is the ideal alternative to dialysis [10]. Furthermore, CRC is a common malignant tumor in Taiwan, with > 15,410 new cases diagnosed in 2013 and an incidence rate of 44.32 per 100,000 [11, 12]. Hence, this nationwide cohort study aimed to assess the CRC risk in KT recipients by reviewing Taiwan’s National Health Insurance Research Database (NHIRD).

## Methods

### Data source

In this study, we retrieved data from the medical claim database of Taiwan’s NHIRD—an extensive database provided by a single-payer, universal, compulsory health care system, National Health Insurance (NHI), for nearly all 23.7 million residents of Taiwan. To date, the NHIRD has been comprehensively used for high-quality epidemiological studies [13, 14] and information on diagnoses, prescriptions, and hospitalizations have been shown to be of good validity [15, 16]. In the present study, we obtained data from the Longitudinal Health Insurance Database (LHID 2000), a subset of NHIRD that comprises historical ambulatory and inpatient care data of 1 million randomly sampled beneficiaries enrolled in the NHI system in 2000. Moreover, the LHID 2000 database has facilitated access to the medical service use history of patients under investigation. Notably, no marked differences were observed in terms of the distributions of age, sex, and health care costs between individuals in the LHID and NHIRD [13, 14].

### Participants

**Fig. 1:**
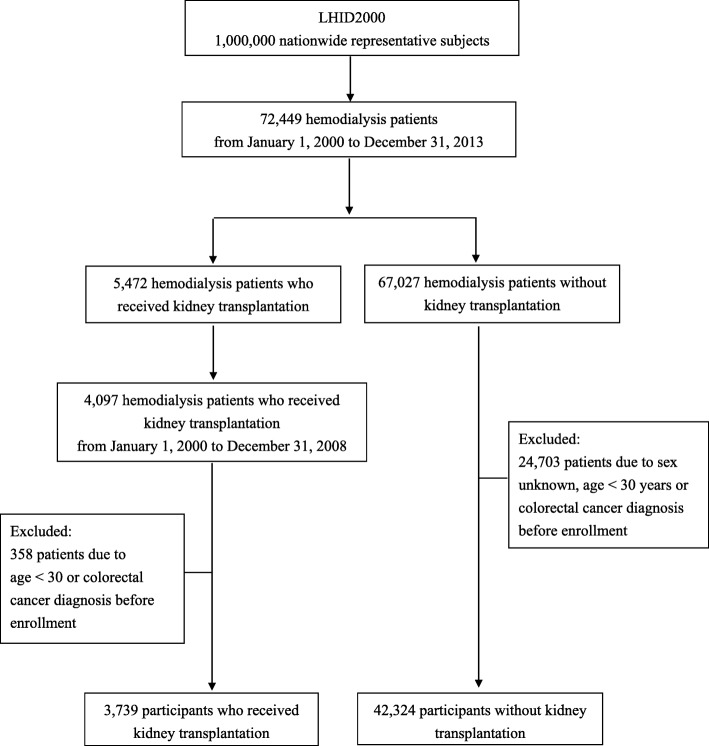
Flow diagram of sample selection *LHID* Longitudinal Health Insurance Database

In this nationwide, retrospective, population-based cohort study, we enrolled patients with a primary diagnosis of ESRD undergoing hemodialysis between January 1, 2000, and December 31, 2008, as per the LHID. This duration was defined as the exposure period to determine hemodialysis patients who received KT. Figure 1 summarizes the subject selection process. First, we identified patients diagnosed with ESRD [International Classification of Disease, Revision 9, Clinical Modification (ICD-9-CM) code: 585] undergoing hemodialysis (ICD-9-CM code: 39.95) and received KT (ICD-9-CM codes: V42.0, 996.81, and V58.69) as the exposed cohort (*n* = 1375). Comparatively, patients with ESRD who were undergoing hemodialysis but not receiving KT were identified as the non-exposed cohort (*n* = 24,703). Patients were excluded if they were younger than 30 years of age, had incomplete demographic data, or were ever diagnosed with CRC prior to the beginning of follow-up (January 1, 2000). Overall, a total of 3739 hemodialytic patients receiving KT (exposed group) and 42,324 not receiving KT (non-exposed group) were included in data analyses.

### Ascertainment of CRC

The primary outcome was the primary diagnosis of CRC (ICD-9-CM codes: 153, 153.0, 153.1, 153.2, 153.3, 153.6, 153.7, 153.8, 153.9154, 154.0, 154.1, 154.2, 154.3, 154.8, and 159.0). In this study, CRC diagnosis was defined according to the Registry for Catastrophic Illness Patient Database (RCIPD), a subpart of the NHIRD. The diagnosis of CRC needs histologic confirmation to be reported in the RCIPD. We followed both cohorts from January 1, 2000, to the date of CRC diagnosis, death (indicated by withdrawal from the NHI), or the end of 2013, whichever occurred first.

### Potential confounders and comorbidities

We noted the covariates that were the potential confounders in the association between KT and CRC. The potential confounders considered in this study included age, sex, and comorbidities, [17] including chronic obstructive pulmonary disease (ICD-9-CM codes: 490, 491, 492, 493, 494, 495, and 496), diabetes mellitus (ICD-9-CM code: 250), coronary artery disease (ICD-9-CM codes: 410, 411, 412, 413, and 414), hypertension (ICD-9-CM codes: 401, 402, 403, 404, and 405), alcohol-related conditions (ICD-9-CM codes: alcoholic liver disease, 571.0, 571.1, 571.2, and 571.3 and alcohol dependence, 303), hypercholesterolemia (ICD-9-CM codes: 272.0, 272.1, 272.2, and 272.4), peptic ulcer (ICD-9-CM codes: 531, 532, and 533), liver cirrhosis and chronic hepatitis (ICD-9-CM code: 571), and inflammatory bowel disease (ICD-9-CM codes: 555 and 556).

### Statistical analysis

We tested differences in descriptive statistics on demographic characteristics and baseline comorbidities between the exposed and non-exposed cohorts using chi-square tests or Student’s *t*-test when appropriate. In addition, we used Kaplan–Meier method to estimate the cumulative incidence of CRC. The log-rank test was performed to assess the difference in the cumulative incidence of CRC between the curves of the cohorts. In addition, we used Cox proportional hazards models to compute hazards ratios (HRs) with 95% confidence intervals (CIs) to determine the association between KT and CRC risk after adjusting for the potential confounders. All statistical analyses were performed using SAS version 9.4 (SAS Institute Inc., Cary, NC), and we set the statistical significance at 0.05 for two-tailed tests.

### Ethics, consent and permission

Since the dataset was released for research purposes and included only scrambled and anonymous information on patient identification, the study was exempt from informed consent from the subjects. Further, the execution of the present study and a waiver of obtaining informed consent from subjects have been approved by the Institutional Review Board of Fu-Jen Catholic University (FJU-IRB NO:C104014).

## Results

Table 1 summarizes the distribution of the baseline demographic characteristics and comorbidities of the cohorts. The mean age (±SD) of the exposed and non-exposed cohorts was 61.84 (±12.63) and 52.31 (±14.74) years, respectively. The exposed cohort was markedly older than the non-exposed cohort. In addition, the exposed cohort had markedly higher proportions of males and comorbidities, including hypertension, hyperlipidemia, diabetes mellitus, coronary artery disease, congestive heart failure, stroke, asthma, and colorectal adenomas, than the non-exposed cohort. However, we observed no significant differences in terms of the proportions of obesity and colorectal polyps between the cohorts.

**Table 1:** Baseline demographics and comorbidities between Kidney transplantation and comparison cohorts

Variable	Kidney transplantation cohort *N* = 3739 (%)	Comparison cohort *N* = 42,324 (%)	*p*-value
Age, years (SD)	61.84 (12.63)	52.31 (14.74)	< 0.001
Sex			< 0.001
Female	2207 (59.03)	23,258 (54.95)	
Male	1532 (40.97)	19,066 (45.05)	
Comorbidity			
Obesity	38 (1.02)	467 (1.10)	0.624
Hypertension	2994 (80.07)	18,245 (43.11)	< 0.001
Diabetes mellitus	1537 (41.11)	9487 (22.42)	< 0.001
Hyperlipidemia	1364 (36.48)	10,448 (24.69)	< 0.001
CAD	1729 (46.24)	10,309 (24.36)	< 0.001
Congestive heart failure	616 (16.47)	2577 (6.09)	< 0.001
Stroke	694 (18.56)	3921 (9.26)	< 0.001
COPD	1030 (27.55)	6230 (14.72)	< 0.001
Asthma	648 (17.33)	4218 (9.97)	< 0.001
Colorectal polyps	33 (0.88)	342 (0.81)	0.627
Colorectal Adenomas	174 (4.65)	1611 (3.81)	0.010

*CAD* Coronary artery disease, *COPD* Chronic obstructive pulmonary disease

**Fig. 2:**
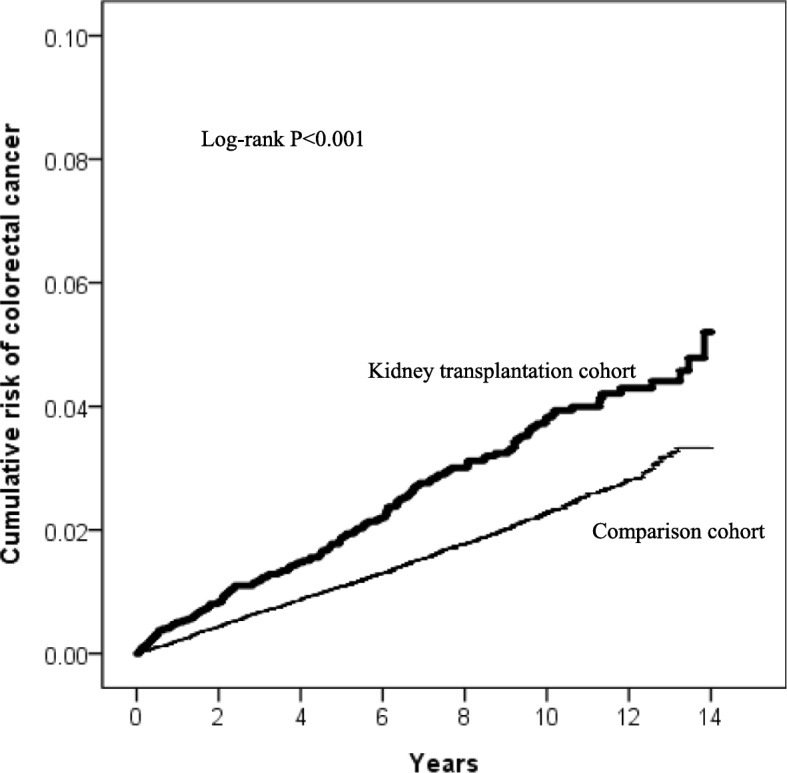
Kaplan-Meier curves for the cumulative risk of colorectal cancer in kidney transplantation and comparison cohorts with the log-rank test

During the follow-up of 36,207 person-years, there were 135 CRC cases in the exposed cohort, resulting in an incidence rate of 372.9 per 100,000 person-years. On the other hand, there were 889 CRC cases in the non-exposed cohort during the follow-up of 3,823,461 person-years, with an incidence rate of 232.5 CRC per 100,000 person-years. Figure 2 presents the Kaplan–Meier curves for the cumulative incidence of CRC for the two cohorts. The log-rank test revealed a significant difference in the cumulative risk of CRC between the cohorts over the entire Kaplan–Meier curve (*P* < 0.001). Notably, the differences were more robust with a prolonged duration of follow-up.

As shown in Table 2, the Cox proportional hazard regression model revealed that KT was significantly associated with an increased risk of CRC after adjusting for potential confounders (adjusted HR, 1.34; 95% CI: 1.11–1.62). Globally, an increase in the risk of CRC related to KT has been observed in both sexes and all age groups. Notably, an elevated risk of CRC related to KT was more evident among women (adjusted HR, 1.48; 95% CI: 1.15–1.91) and those aged < 50 years (adjusted HR, 2.08; 95% CI: 1.16–3.73).

**Table 2:** Multivariate associations between kidney transplantation and colorectal cancer (CRC) incidence among transplant recipients, overall and by age and sex

Variable	Kidney transplantation cohort			Comparison cohort			HR (95% CI)	Adjusted HR (95% CI)
	No. CRC	PYs	Rate	No. CRC	PYs	Rate		
Overall	135	36,207	372.9	889	382,346	232.5	1.59 (1.33–1.91)	1.34 (1.11–1.62)
Age group								
< 50	13	7225	179.9	198	187,138	105.8	1.64 (0.93–2.90)	2.08 (1.16–3.73)
50–59	27	8098	333.4	202	79,980	252.6	1.27 (0.85–1.90)	1.50 (0.99–2.28)
60–69	45	10,991	409.4	244	67,386	362.1	1.12 (0.82–1.54)	1.35 (0.97–1.87)
≧70	50	9893	505.4	245	52,841	463.7	1.12 (0.82–1.50)	1.23 (0.90–1.67)
Sex								
Female	77	21,538	357.5	388	209,120	185.5	1.91 (1.50–2.44)	1.48 (1.15–1.91)
Male	58	14,669	395.4	501	173,226	289.2	1.36 (1.04–1.78)	1.20 (0.90–1.58)

*PYs* Person-years, *HR* Hazard ratio, *CI* Confidence interval

Rate: incidence rate per 100,000 person-years

Hazard ratios were adjusted for age, sex, and comorbidities, including obesity, hypertension, diabetes, hyperlipidemia, coronary artery disease, congestive heart failure, stroke, chronic obstructive pulmonary disease, asthma, colorectal polyps, and colorectal adenomas

## Discussion

This nationwide, retrospective, population-based cohort study established that hemodialytic patients undergoing KT exhibit a markedly higher risk of CRC than those not undergoing KT. After adjusting for potential confounders, KT recipients had a 1.34-fold increased risk of CRC as compared with those not undergoing KT.

The introduction of potent immunosuppressive agents offered prolonged survival to transplant recipients, facilitating the documentation of an increased incidence of malignancies in this population [1–7, 18–21]. Thus, malignancies constitute a significant cause of late morbidity and mortality in renal allograft recipients. Compared with the general population, accumulating evidence suggests a higher risk of CRC in solid organ transplant recipients [18–23] and KT recipients [1–7]. The overall risk of CRC has increased by, at least, 2- to 2.5-fold in the kidney transplant population, and the pattern of elevated risk seems to be the highest in younger patients [1–7]. In a previous study, SIR for younger patients was 13.5, falling to < 3 for older patients aged ≥55 years compared with the age- and sex-matched general population [24]. Correspondingly, this study suggested that hemodialytic patients undergoing KT exhibit a markedly elevated risk of CRC compared with those not undergoing KT. In addition, the pattern of elevated risk was more pronounced among younger and female hemodialytic patients. Reportedly, immunosuppression following transplantation could be a critical factor in the amplified incidence of malignancy [6, 7]. Some studies have suggested that immunosuppression regimen biologically promotes systemic inflammation, resulting in immune dysregulation, increased DNA damage by upregulating the expression of tumor growth factor-β and vascular endothelial growth factor, and altered gut microbiota, which are conducive to colorectal tumorigenesis [25, 26]. Conversely, a recent meta-analysis of cancer incidence including dialysis-dependent patients and KT recipients established no correlation between transplantation and CRC development [27]. These disparities necessitate comprehensive studies to elucidate the risk of KT-associated CRC.

The findings of this study should be interpreted within the context of some limitations. Notably, studies based on insurance claims or other third-party data are often flawed because the information on confounding factors in insurance data is often limited [16, 28]. In this study, the information on major confounders, such as family history of CRC, obesity, smoking habits, and dietary patterns, was not available in the NHIRD. Thus, this study has residual confounding in the assessment of KT-associated CRC risk. Furthermore, the physical function before and after KT in hemodialytic patients was not assessed in the NHIRD.

Nevertheless, this study has some strengths. It is a national cohort study based on Taiwan’s NHIRD, which contains data from Taiwan’s compulsory and universal health care system that has a high coverage rate in Taiwan, facilitating analysis in a real-life setting of an unselected patient population. Furthermore, patient dropout was avoided, and selection bias minimized owing to the use of routine database records.

## Conclusions

In conclusion, this study demonstrated that the CRC incidence in hemodialytic patients undergoing KT markedly increased compared with hemodialytic patients not undergoing KT. In addition, this study and biological plausibility support the association between KT and CRC. Although further research is warranted, the emerging data suggest that screening colonoscopy before and after transplantation will result in earlier detection of CRC, possibly decreasing mortality in this population. Indeed, according to the National Comprehensive Cancer Network (NCCN) guideline for colonoscopic negative/no polyps patients, the next colonoscopy should be recommended every 5–10 years [29]. However, routine CRC screening recommendations, such as colonoscopy, stool DNA tests or other stool based tests, may need to be revised after KT since individuals getting a transplant during the shorter follow-up period.

## Data Availability

With strict confidentiality guidelines being closely followed in accordance with personal electronic data protection regulations in Taiwan, the datasets used in the current study are not available from request.

## Abbreviations

CI: Confidence interval
CRC: Colorectal cancer
ESRD: End-stage renal disease
HR: Hazards ratio
ICD-9-CM: International Classification of Disease, Revision 9, Clinical Modification
KT: Kidney transplantation
LHID: Longitudinal Health Insurance Database
NCCN: National Comprehensive Cancer Network
NHI: National Health Insurance
NHIRD: National Health Insurance Research Database
NHRI: National Health Research Institute
RCIPD: Registry for Catastrophic Illness Patient Database
SIR: Standardized incidence ratio
